# Dual‐axis rotational coronary angiography can reduce peak skin dose and scattered dose: a phantom study

**DOI:** 10.1120/jacmp.v15i4.4805

**Published:** 2014-07-08

**Authors:** Huiliang Liu, Zhigeng Jin, Yunpeng Deng, Limin Jing

**Affiliations:** ^1^ Department of Cardiology General Hospital of Chinese People's Armed Police Forces Beijing China

**Keywords:** coronary angiography, rotational coronary angiography, dual‐axis angiography, radiation dose, patient dose, staff dose

## Abstract

The purpose of this study was to evaluate peak skin dose received by the patient and scattered dose to the operator during dual‐axis rotational coronary angiography (DARCA), and to compare with those of standard coronary angiography (SA). An anthropomorphic phantom was used to simulate a patient undergoing diagnostic coronary angiography. Cine imaging was applied on the phantom for 2 s, 3 s, and 5 s in SA projections to mimic clinical situations with normal vessels, and uncomplicated and complicated coronary lesions. DARCA was performed in two curved trajectories around the phantom. During both SA and DARCA, peak skin dose was measured with thermoluminescent dosimeter arrays and scattered dose with a dosimeter at predefined height (approximately at the level of left eye) at the operator's location. Compared to SA, DARCA was found lower in both peak skin dose (range: 44%–82%, *p* < 0.001) and scattered dose (range: 40%–70%, *p* < 0.001). The maximal reductions were observed in the set mimicking complicated lesion examinations (82% reduction for peak skin dose, *p* < 0.001; 70% reduction for scattered dose, *p* < 0.001). DARCA reduces both peak skin dose and scattered dose in comparison to SA. The benefit of radiation dose reduction could be especially significant in complicated lesion examinations due to large reduction in X‐ray exposure time. The use of DARCA could, therefore, be recommended in clinical practice to minimize radiation dose.

PACS numbers: 87.53.‐j, 87.53.Bn, 87.59.‐e, 87.59.C‐, 87.59.cf, 87.59.Dj

## I. INTRODUCTION

As interventional cardiac procedures become indispensable in the present cardiology practice, concerns arise over potential radiation risk to both patients and operators.^1^, ^2^ There are two types of biological effects of radiation: stochastic effects and tissue reactions (previously called deterministic effects).^(3)^ Tissue reactions are caused by radiation dose that exceeds specific thresholds. Typical examples of tissue reactions of cardiological interest are cataract formation (in operators) and skin injury (in patients). Note that data from human populations under radiation exposure suggest that cataract occurs at doses far lower than the previous consensus.^(4)^ Moreover, there has been an increasing number of case reports on patient skin injuries resulting from interventional procedures.^5^, ^6^, ^7^ Thus, in order to prevent serious tissue reactions in clinical practice, actions to control radiation dose need to be taken.

Recently, dual‐axis rotational coronary angiography (DARCA) has developed as an innovative adaptation to the standard coronary angiography (SA), which remains the gold standard for assessing coronary stenosis.^(8)^ During DARCA, the three‐dimensional rotation of the gantry around the patient occurs in the left anterior oblique (LAO)/right anterior oblique (RAO) and cranial/caudal orientations during one cine acquisition, to obtain images in a trajectory specifically designed to reduce vessel foreshortening. Compared to SA, this creative technology demonstrates significant potential in reducing patient radiation dose, contrast utilization, and procedure time, while enhancing the number of angiographic projections obtainable with superior imaging results.^8^, ^9^, ^10^, ^11^ However, the above‐mentioned dose reductions have been reported with dose area product (DAP), which gives no direct information on peak skin dose (PSD). ^12^, ^13^, ^14^, ^15^, ^16^ Moreover, estimating skin dose from DAP has a potential error of at least 30%–40%.^(17)^ The impact of DARCA on patient skin dose, compared with SA, remains unknown. In addition, as far as we know, there has been no study on the scattered dose during DARCA compared to SA. The purpose of this study was to evaluate patient PSD and scattered dose to the operator in DARCA compared to SA with the use of an anthropomorphic phantom.

## II. MATERIALS AND METHODS

### A. Coronary angiography

**Figure 1:**
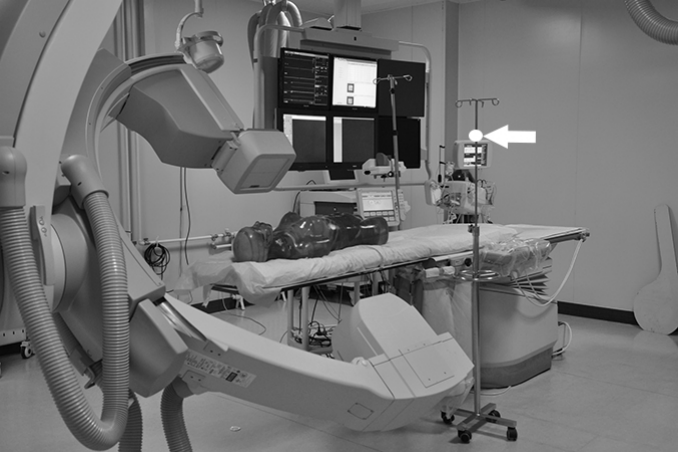
Coronary angiography performed on an anthropomorphic phantom. For scattered dose measurements, the dosimeter was clamped to a drip stand at the height of 170 cm (white arrow), which was positioned at a distance of 91 cm from the exposure center of phantom. No radiation protections were used during the measurements.

This study was conducted in the cardiac catheterization laboratory of a tertiary referral center using a monoplane AlluraXper FD10 fluoroscopy device (Philips Healthcare, Best, The Netherlands). Diagnostic coronary angiography was simulated on an anthropomorphic female phantom (ART‐200; RGRMS, China) by an experienced interventional cardiologist (Fig. 1). Exposure parameters (e.g., X‐ray tube voltage and tube current) were set by the X‐ray system's automatic exposure control.

SA projections according to our routine coronarography were used. Left coronary angiography was performed with five exposures in five projections: $\text{RAO}\;25^{\circ }/\text{caudal}\;25^{\circ },\;\text{RAO}\;25^{\circ }/\text{cranial}\;25^{\circ },\;\text{LAO}\;45^{\circ }/\text{cranial}\;25^{\circ },\;\text{LAO}\;45^{\circ }/\text{caudal}\;25^{\circ }$, and anterior‐posterior $(\text{AP})/\text{cranial}\;25^{\circ }$. Right coronary angiography was done with two exposures in two projections: LAO 45° and AP/cranial 25°. As the complexity of individual lesions can influence the SA exposure time to the patient, based on our center's image study averages, we applied 2, 3, and 5 s cine imaging in each projection to represent clinical situations with normal, uncomplicated, and complicated vessels, respectively. Moreover, a 3 s fluoroscopy in each projection was included for table panning to position the region of interest to mimic clinical practice. Therefore, each exposure consisted of 3 s fluoroscopy and 2, 3, or 5 s cine imaging.

Prior to DARCA, isocentering of the imaged coronary arteries is essential. The technique was described in a previous study.^(10)^ To simulate the workflow, separate 5 s fluoroscopy runs were acquired at AP and LAO 45° projections before a left coronary DARCA, and at AP projection before a right coronary DARCA. Cine time required for left and right coronary DARCA averages 6.8 s and 4.5 s, respectively. Detailed X‐ray exposure factors for the SA and DARCA are shown in Table 1.

**Table 1:** X‐ray exposure factors for different coronary angiographic protocol

*Exposure Factors*	*2 s SA*	*3 s SA*	*5 s SA*	*DARCA*
Fluoroscopy time per run (s)	3	3	3	5
Cine time per run (s)	2	3	5	6.8 (4.5)^a^
Total fluoroscopy time (s)	21	21	21	15
Total cine time (s)	14	21	35	11.3
Field of view (cm)	25	25	25	25
Source‐image detector distance (cm)	100	100	100	120
Source‐skin distance (cm)	60	60	60	60
Fluoroscopy mode	Low	Low	Low	Low
Cine mode (fr/s)	15	15	15	15

a Number in the bracket indicates cine time for the right coronary spin; out of the bracket for the left coronary spin.

SA = standard coronary angiography; DARCA = dual‐axis rotational coronary angiography.

### B. Dosimetric measurements

**Figure 2:**
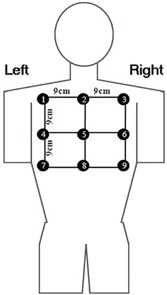
The thermoluminescent dosimeters (TLDs) positions used in the skin dose measurement. The TLD array was placed under the upper back of the anthropomorphic phantom, beneath the heart. TLDs were spaced by 9 cm from each other and numbered by 1 to 9. TLD = thermoluminescent dosimeter.

PSD was measured by thermoluminescent dosimeter (TLD) arrays (Harshaw TLD‐100 chips) attached to the phantom's upper back (Fig. 2). For each measurement site, three TLDs wrapped in black nylon film were irradiated, with the average signal being determined. An extra set of three TLDs was kept outside the radiation room to obtain background readings. The TLDs were read out using a Harshaw 3500 TLD reader (Thermo Fisher Scientific Inc, Waltham, MA). The maximum TLD reading was regarded as the PSD. In addition, each angiography was performed five times to obtain sufficient exposure to the TLDs, and the total dose was divided by five. A preselected group of TLDs was used with sensitivity variation ±3% to minimize variability. The calibration of the TLDs was traceable to the National Standard Dosimetric Laboratory. The overall uncertainty of TLD readings was less than 5%. Moreover, DAP, cumulative air kerma (CAK), and dose rate were registered from the X‐ray system. Then, dose indexes (the ratio between PSD and CAK) were calculated per measurement.^(18)^

Scattered dose to the operator was measured with no shielding in place by an electronic personal dosimeter (Unfors EDD‐30; Unfors Instruments AB, Billdal, Sweden). This detector has an accuracy of ±6% and an ample dynamic range of dose rates between 0.03 mSv/h and 2 Sv/h. To simulate clinical angiography procedures by radial access, the dosimeter was clamped to a drip stand, which was laterally positioned at 44 cm from the phantom center, at a height of 170 cm (approximately at the operator's left eye, Fig. 1). The dosimeter was about 91 cm from the phantom center.

In addition, we evaluated the influence of source‐image detector distance (SID) (100, 110, and 120 cm) on fluoroscopy entrance dose rate and cine entrance dose per frame at the patient entrance reference point in AP projection using low fluoroscopy mode and 15 fr/s cine with 25 cm field of view. To ensure high reliability of the results, all the same experiments were repeated ten times.

### C. Statistical analysis

All measurement results were expressed as mean ± SD and tested with repeated measures analysis of variance with post hoc Student's *t*‐test using SPSS (Version 19.0, SPSS Inc., Chicago, IL). A p‐value of <0.05 was considered statistically significant.

## III. RESULTS

### A. Patient dose

As shown in Table 2, PSD (maximum TLD readings), DAP, and CAK were generally lower in DARCA than in SA. DARCA accounted for 44% less PSD(p=0.004), 27% less DAP(p=0.006), and 25% less CAK(p=0.004) than 2 s SA. When compared to 3 s and 5 s SA, DARCA corresponded to 59% and 82% lower PSD, 52% and 76% lower DAP, and 43% and 74% lower CAK, respectively (all p<0.001). Note, the minimum TLD measurement during DARCA was higher than during 2 s SA (p<0.001), while the mean TLD results remained comparable (p>0.05). In addition, DARCA was associated with a smaller dose index in comparison to SA (all p<0.05).

**Table 2:** Radiation dose to the phantom patient during coronary angiography.^a^

*Coronary Angiography*	*TLD (mGy)*			*DAP* $\left(Gy\times cm^{2}\right)$	*CAK (mGy)*	
	*Min*	*Max*	*Mean*			*Dose Index*
2 s SA	0.71±0.05	12.12±0.25	3.42±0.02	4.21±0.21	85.41±3.29	0.14±0.01
3 s SA	1.33±0.08	16.47±1.17	5.60±0.03	6.43±0.35	112.19±6.12	0.15±0.02
5 s SA	1.60±0.06	36.99±2.03	9.23±0.47	12.81±0.32	243.09±5.84	0.16±0.01
DARCA	1.37±0.08	6.80±0.42	3.69±0.03	3.06±0.21	64.00±3.69	0.10±0.01

a Values are presented as mean ± SD.

SA=standard coronary angiography; DARCA=dual‐axis rotational coronary angiography; TLD=thermoluminescent dosimeter; DAP=dose area product; CAK=cumulative air kerma.

**Figure 3:**
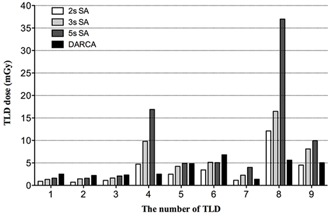
Skin dose distribution in the thermoluminescent dosimeter arrays separated by different angiographic protocol. 2 s, 3 s, and 5 s cine time was applied in each projection during the 2 s, 3 s, and 5 s SA, which represents evaluating normal vessels, uncomplicated, and complicated coronary lesions in clinical practice, respectively. TLD = thermoluminescent dosimeter; SA = standard coronary angiography; DARCA = dual‐axis rotational coronary angiography.

Skin dose distribution was plotted in Fig. 3 per TLD measurement and per angiographic protocol. During SA, the most irradiated TLDs were No. 4, 5, 6, 8, and 9, among which No. 8 received the highest dose. During DARCA, the most irradiated TLDs were No. 5, 6, 8, and 9, with No. 6 exposed to the highest dose.

### B. Operator dose

Table 3 shows a comparison of scattered dose to the operator. DARCA was found to reduce 40%, 53%, and 70% of scattered dose than that in 2 s, 3 s, and 5 s SAs, respectively (all p<0.001).

**Table 3:** Scattered dose to the operator during coronary angiography.^a^

*Coronary Angiography*	*Dose in Fluoroscopy* (μSv)	*Dose in Cine* (μSv)	*Total Dose* (μSv)
2 s SA	2.45±0.02	8.00±0.10	10.45±0.08
3 s SA	2.44±0.04	10.88±0.17	13.32±0.17
5 s SA	2.48±0.05	18.85±0.26	21.32±0.28
DARCA	1.75±0.05	4.55±0.04	6.31±0.07

a Values are presented as mean ± SD.

SA=standard coronary angiography; DARCA=dual‐axis rotational coronary angiography.

### C. The influence of SID on entrance dose rate and dose per frame

Entrance dose rate in fluoroscopy and dose per frame in cine both significantly increased with greater SID (p<0.001, Table 4).

**Table 4:** Entrance dose rate during fluoroscopy and dose per frame during cine for different source‐image detector distances.^a^

*Source—image Detector Distance (cm)*	*Fluoroscopy Dose Rate* (mGy/min)	*Cine Dose per Frame* (μ Gy/fr)
100	13.04±0.22	214.67±2.52
110	16.09±0.24	266.00±2.00
120	19.69±0.80	318.00±3.00

a Entrance dose rate and dose per frame were measured at the patient entrance reference point in the anterior‐posterior projection (in the low fluoroscopy mode with 25 cm field of view and 15 fr/s for both fluoroscopy and cine)

## IV. DISCUSSION

The present study has used an anthropomorphic phantom for radiation dose measurements during coronary angiography simulations. It allows a controlled measurement approach to avoid clinical variabilities (e.g., patient differences or clinical procedural factors), and, therefore, results in a reliable dosimetry assessment of DARCA and SA. To our knowledge, this is the first study that focuses on PSD and scattered dose in DARCA in comparison to SA.

This study demonstrates that the PSD during DARCA is significantly lower than that during SA. Several factors may contribute to lower the PSD. During DARCA exams, less fluoroscopy time and cine frames are needed than in SA. This results in considerably lower DAP and CAK (i.e., much less radiation energy) to the patient. Moreover, the results show that using DARCA leads to a lower dose index than in SA. This, in turn, indicates that DARCA is more effective in reducing PSD, as it could spread the administered radiation over a large skin area.

A major finding of this work is that complicated lesion examinations could benefit the most from PSD reduction with DARCA. This is of practical importance. Patients with complicated lesions are likely to undergo reoccurring invasive treatments in the near future because of disease recurrence. The limits of skin tolerance to radiation may then be exceeded, resulting in skin injuries.^(19)^ By applying DARCA instead of SA, the risk could be lowered. While most of coronary angiography procedures have a PSD well below the 2 Gy threshold for skin injuries (early transient erythema),^(3)^ procedures that use ionizing radiation should be performed in accordance with the As Low As Reasonably Achievable (ALARA) philosophy. Even when the skin dose is below the threshold, the probability of stochastic effects shall not be neglected. In addition, although 2 Gy is the threshold to cause the earliest detectable effect on the skin, the actual radiation needed to cause injury varies, depending on individual biological sensitivity and the presence of coexisting diseases (e.g., diabetes mellitus and connective tissue disorders).^3^, ^20^ For this reason, some patients could show signs of deterministic injury at a relatively low dose. Moreover, sensitive patients are likely to experience more severe injury than typical patients at equal doses. Thus, it is of great relevance to apply DARCA for lowering patient radiation.

Compared to the 2 s SA (i.e., angiography of normal vessels), DARCA seems to have a higher minimum skin dose despite less exposure time and a comparable mean skin dose. Figure 3 shows that, except for No. 4 and No. 8 TLDs, all the other TLD readings are higher in DARCA than in 2 s SA. It suggests that during DARCA, the TLDs (except No. 4 and No. 8) may have more incidences of being irradiated due to more imaging projections than in SA, resulting in a higher minimum TLD reading. We also observed that, although the skin dose distribution on the body surface is different between DARCA and SA, the most irradiated body area is similar, owing to all the traditional coronary angiographic views are encompassed by the trajectories of DARCA.^(10)^

Note that a greater SID is used during DARCA compared to SA (120 cm vs. 100 cm). This, in principle, would lead to higher entrance dose rate or dose per frame to the patient (i.e., 51% and 48% for fluoroscopy and cine, respectively). However, the exposure time for the SA is much longer than that for the DARCA (fluoroscopy: 21 s vs. 15 s; cine: 23.3 s (average) vs. 11.3 s). The relative increases in radiation dose caused by the longer exposure time during SA are 40% and 106% for fluoroscopy and cine, respectively. As cine imaging contributes more to the total amount of radiation dose during diagnostic coronary angiography because of a higher dose rate,^(21)^ a lower total radiation dose is expected in DARCA, even with a greater SID.

The lens of the eye is one of the most radiosensitive tissues in the body. Exposure of the lens to ionizing radiation can cause cataract at approximately 2 Gy.^(22)^ Recent studies have indicated an even lower threshold in the order of 0.5 Gy, or that risk increases linearly with radiation dose, with no apparent threshold.^4^, ^23^ Moreover, a prior study has found a high percentage of lens opacities in interventional cardiologists attributable to occupational radiation exposure. ^(24)^ Thus, there is an urgent need to apply various radiation protection strategies to reduce the risk of cataract formation. The present study indicates that scattered dose at the level of the operator's left eye during DARCA is significantly lower than during SA, particularly while examining complicated lesions. As commonly agreed, staff dose is largely related to patient dose since it results from secondary scattered radiation arising mainly from the patient. We can infer that scattered dose reduction would also exist in the other sites of the operator's body, as a lower patient radiation dose is associated with DARCA when compared to SA. This would have a significant impact on the annual dosage for the operators, especially for the high‐volume operators. Therefore, this new imaging technique could be used widely for assessing coronary stenosis, in order to reduce the radiation doses both to the patient and the operator. Note that this study may benefit from a greater range of positional variation in the operator dose measurements due to the inherent differences in beam projections. However, this effect may be limited. The reasons are as follows. For one thing, all the traditional coronary angiographic views are encompassed by the trajectories of DARCA. For another, the DAP‐normalized operator dose $\left(\mu \text{Sv}/\text{Gy}\times {\text{cm}}^{2}\right)$ (operator dose divided by the DAP), which is a reliable estimation of local scattered dose by simple documentation of DAP,^(25)^ was comparable between SA and DARCA in the present study (mean 1.92 for SA and 2.06 for DARCA). Accordingly, the lower scattered dose associated with DARCA in this study was mainly as a result of smaller patient radiation exposure during DARCA.

We should pay attention to the fact that both patient and staff doses noted in this study are markedly lower than those in clinical practice described previously. For SA, in the study of van de Putte et al.,^(12)^ they have reported higher DAP and PSD values ($61\;\text{Gy}\times {\text{cm}}^{2}$ for DAP and 412 mGy for PSD) than those in the present study (average: $8\;\text{Gy}\times {\text{cm}}^{2}$ and 22 mGy, respectively). Recent studies have reported $21\;\text{Gy}\times {\text{cm}}^{2}$ and 160 mGy for DAP and PSD, respectively. ^10^, ^16^ Moreover, Politi et al.^(26)^ have reported that the operator's left eye exposure was 297 μSv during transradial SA, which was significantly greater than that in our study (average: 15 μSv). For DARCA, the DAP ranged from 9 to $24\;\text{Gy}\times {\text{cm}}^{2}$.^10^, ^11^ However, to the best of our knowledge, no data were available for PSD and scattered dose during DARCA. These lower radiation exposures can be explained by the following reasons. First, the anthropomorphic phantom used in our study is without arms and legs, and the body mass index of this phantom is about 21. It cannot represent the average cardiac patients in clinical practice who usually have larger values of body mass index,^1^, ^10^ which is positively correlated with radiation dose.^(10)^ In addition, phantom studies tend to underestimate the operator's radiation exposure, since movements of the personnel during examinations are difficult to simulate.^(27)^ Second, the radiation required to advance the catheter across the aortic arch, engage the coronary ostia, exchange catheters, and perform noncoronary angiography were excluded from the analysis in this study, because they are confounding factors for the comparison of radiation dose between the two coronary angiography modalities. Third, as insufficient experience in performing the procedure and radiation protection techniques can result in a higher radiation dose,^(28)^ this phantom study had eliminated these factors from the analysis. Finally, the additional coronary acquisitions are usually applied in clinical practice at the discretion of the operator beyond the standard angiography protocol. This study has used the standard angiography protocols only, which are routinely applied in our catheterization lab. Although the patient radiation dose noted in this study is obviously lower than that in clinical practice, the prior study has reported a mean DAP value of $7\;\text{Gy}\times {\text{cm}}^{2}$ when optimized radiation protection techniques are used for SA,^(29)^ which is at similar level as reported in this study. To some extent, the results of this study may represent the differences between the two techniques in this context.

The present study is limited by a few factors. Firstly, our study was performed in a simulated cardiac catheterization lab environment, and cannot fully account for the variables and conditions present in clinical practice. Secondly, as mentioned above, the anthropomorphic phantom used in this study is lacking arms and legs and is of small body mass index. The morphology of a real patient is not fully resembled. Smaller DAP is also expected here when compared with values from average cardiac patients with larger body mass index. Moreover, phantom studies tend to underestimate the operator's radiation exposure. Thirdly, it is well known that PSD is not easy to measure. With the number of TLDs and measurement points used in our study, potentially higher skin doses might not have been detected. With larger numbers of TLDs, further investigations could be more accurately done on patient skin dose distribution. Finally, operator position may vary between centers during the procedure, which could influence individual radiation exposure. During our study, the distance to the X‐ray source was fixed and, therefore, the measured dosage should be judged as an average value. In general, although the above measurements might be distinct from the actual radiation doses, the results in relative reductions between SA and DARCA are still credible and evident.

## V. CONCLUSIONS

The present study demonstrates that, compared to SA, DARCA can reduce 44%–82% of PSD to the patient and 40%–70% of scattered dose to the operator. The benefit of radiation dose reduction could be especially significant in complicated lesion examinations due to large reduction in X‐ray exposure time. The use of DARCA could, therefore, be recommended in clinical practice to minimize the patient radiation dose and staff dose.

## ACKNOWLEDGMENTS

We gratefully thank the following people in National Institute for Radiological Protection of Chinese Center for Disease Control and Prevention: Kedao Wei and Jianchao Wang, for access to their facilities and help with the measurements.
